# Update on thyroid-associated Ophthalmopathy with a special emphasis on the ocular surface

**DOI:** 10.1186/s40842-016-0037-5

**Published:** 2016-11-16

**Authors:** Priscila Novaes, Ana Beatriz Diniz Grisolia, Terry J. Smith

**Affiliations:** 1 Department of Ophthalmology and Visual Sciences, Kellogg Eye Center, University of Michigan Medical School, Ann Arbor, MI 48105 USA; 2 Division of Metabolism, Endocrinology, and Diabetes, Department of Internal Medicine, University of Michigan Medical School, Ann Arbor, MI 48105 USA; 3 Department of Ophthalmology and Visual Sciences, Brehm Tower, Room 7112, 1000 Wall Street, Ann Arbor, MI 48105 USA

**Keywords:** Graves’ disease, Ophthalmopathy, Thyroid, Orbit, Ocular surface

## Abstract

Thyroid-associated ophthalmopathy (TAO) is a condition associated with a wide spectrum of ocular changes, usually in the context of the autoimmune syndrome, Graves’ disease. In this topical review, we attempted to provide a roadmap of the recent advances in current understanding the pathogenesis of TAO, important aspects of its clinical presentation, its impact on the ocular surface, describe the tissue abnormalities frequently encountered, and describe how TAO is managed today. We also briefly review how increased understanding of the disease should culminate in improved therapies for patients with this vexing condition.

## Background

Thyroid-associated ophthalmopathy (TAO^a^, aka thyroid eye disease or Graves’ ophthalmopathy) refers to several ocular manifestations related to the systemic autoimmune process, Graves’ disease (GD) [1]. This syndrome has been attributed to the loss of immune tolerance to the thyrotropin receptor (TSHR) and perhaps other auto-antigenic proteins [2, 3]. TAO results from the linked conspiracy of auto-reactivity and tissue remodeling. The factors that over-arch the ocular components of GD with the pathology occurring in the thyroid have yet to be identified unambiguously. TAO is the most common and serious extra-thyroidal manifestation of GD, with 25–50% in those with the thyroid disease [4–6]. While the majority of individuals with GD become hyperthyroid sometime in the course of their disease, TAO can also occur in primary hypothyroidism and in patients who remain euthyroid [7]. Substantial evidence suggests that GD and TAO result from complex interplay between genetic susceptibility, epigenetic factors, and several partially characterized environmental triggers [8]. Several recent reviews have addressed TAO; however none has detailed insights of the association and interactions between TAO, the ocular surface (OS) and dry eye syndrome (DES). We have thus included an emphasis on the OS and DES in this review.

## Pathogenesis

The central participant in the pathogenesis of TAO is the orbital fibroblast [9, 10]. Orbital fibroblasts from healthy individuals appear to differ from those with TAO [11–13]. They represent a heterogeneous population of cells with divergent capacities for terminal differentiation and gene expression. We now know that a subpopulation of orbital fibroblasts in TAO derive from bone marrow derived fibrocytes [14]. These cells express several thyroid antigens that have been implicated in TAO. Among them is the TSHR. TSHR has been detected in orbital connective tissue and on orbital fibroblasts, albeit at extremely low levels [15, 16] but the basis for its expression has remained uncertain. Fibrocytes were found to infiltrate the TAO orbit and express higher levels of TSHR than those found on orbital fibroblasts [14, 17, 18]. Furthermore, the receptor displayed on fibrocytes is functional in that TSH and thyroid-stimulating immunoglobulins (TSI) provoke the generation of extremely high levels of inflammatory cytokines. These include IL-6, TNF-α, IL-8, and IL-1β [19]. How the receptor participates in TAO is less certain. In addition to TSHR, other thyroid autoantigens have also been detected in orbital tissues and expressed by orbital fibroblasts [8]. Persistence of detectable thyroid autoantibodies in patients with therapy-resistant TAO may support a role for these autoantigens in disease pathogenesis [20].

It is unclear how the abnormal behavior of orbital fibroblasts in TAO interplays with the recruited lymphocytes, mast cells and macrophages. Their accumulation in the orbit is characteristic of the disease. Development of their therapeutic targeting is of considerable importance [12, 18]. Subpopulations of orbital fibroblasts may explain, at least in part, the diversity of clinical TAO presentation. The disease can manifest as predominantly fat expansion or with isolated extraocular muscle (EOM) involvement or most commonly a mixture of both [13, 21]. Fibroblasts can be identified on the basis of cell surface markers such as Thy-1 and CD34. Subsets differ in their ability to differentiate into adipocytes or myofibroblasts [13, 22]. Cells from orbital fat differ phenotypically from those of perimysial derivation [23].

TAO is associated with accelerated glycosaminoglycan production, resulting in mechanical embarrassment of the orbital contents [14, 24, 25]. Orbital fibroblast proliferation and differentiation into adipocytes leads to fat tissue expansion. In muscle, increased glycosaminoglycan accumulation can interfere with normal contraction and movement [9, 14, 25]. In advanced stages of the disease, fibrotic changes can affect muscle functions, resulting in restricted eye movement.

Many clinical signs and symptoms of TAO arise from expansion of soft-tissues within the orbit, leading to exophthalmos [9, 25, 26]. Disturbance of the ocular surface is caused by inadequate lid coverage; the increased palpebral fissure width results in accelerated tear evaporation and elevated tear osmolarity [27, 28], perpetuating an inflammatory cycle [29] and contributing to a major source of disease morbidity.

Eyelid structures are also altered in TAO, resulting in retraction of both upper and lower eyelids. Three different mechanisms have been proposed [24]. The cicatricial and restrictive theory is explained by the effects of TAO on EOM and on the elastic components of eyelid retractor muscles. Enlarged inferior rectus muscle dimension and the generalized orbit connective tissue congestion may retract the lower eyelid margin. This is due to increased tension on the lower eyelid complex (inferior oblique muscle, inferior rectus muscle and the capsulopalpebral fascia (CPF). In the upper eyelid, fibrosis of the Müller and *levator palpebrae superioris* muscles (LPS) is variable. Muscle hyperaction results from increased sympathetic stimulation of the inferior tarsal muscle (lower eyelid) or Müller’s muscle (upper eyelid). This is thought to be a consequence of direct thyroid hormone actions. The concept of anterior globe displacement as a mechanism for eyelid retraction resulting from proptosis is demonstrated by improvement of eyelid retraction following surgical correction of proptosis [24, 30, 31]. Overall, these theories appear compatible with the variations of clinical disease presentation.

## Clinical presentation

The onset of ocular symptoms/signs and hyperthyroidism can occur simultaneously or diverge temporally by months to years [32]. Patients undergo an initial period where inflammation, progressive orbital congestion, and variably worsening proptosis evolve. This stage is termed the active phase. The activity of the disease can be assessed by ﻿calculating the clinical activity score (CAS), based on seven signs (Table 1). In addition, clinical severity can be classified using the NOSPECS score (Table 2)﻿. According to Rundle’s curve, this phase can last from months to several years [33]. Activity gives way to a period of stabilization and ultimately leads to the inactive phase where the disease no longer progresses. This stable phase is seldom associated with a complete normalization of ocular changes [4].

**Table 1:** Clinical activity score

GO activity (CAS)	
1	Spontaneous retrobulbar pain
2	Pain on attempted upward or downward gaze
3	Redness of eyelids
4	Redness of conjunctiva
5	Swelling of caruncle or plica
6	Swelling of eyelids
7	Swelling of conjunctiva (chemosis)

**Table 2:** TAO Eye changes classification - NOSPECS

NOSPECS		
Class	Grade	Criteria
1		No physical signs or symptoms
		Only signs (limited to upper lid retraction, stare, and lid lag)
2		Soft tissue involvement (with symptoms and signs)
	0	Absent
	a	Minimal
	b	Moderate
	c	Marked
3		Proptosis ≥3 mm above upper normal limit
	0	Absent
	a	3–4 mm increase over upper normal
	b	5–7 mm increase
	c	≥8 mm increase
4		Extraocular muscle involvement
	0	Absent
	a	Limitation of motion extremes of gaze
	b	Evident restriction of motion
	c	Fixation of a globe or globes
5		Corneal involvement
	0	Absent
	a	Stippling of cornea
	b	Ulceration
	c	Clouding, necrosis, perforation
6		Sight loss (optic nerve involvement)
	0	Absent
	a	Disc pallor or visual field deffect; vision 20/20–20/60
	b	Same as 6﻿a, but vision 20/70–20/200
	c	Blindness, i.e., failure to perceive light, vision < 20/200

Proptosis or exophthalmos occurs as a consequence of expanding orbital contents being confined within the boney orbit and the naturally occurring decompression resulting from anterior displacement of the globe. TAO is the most common cause of both unilateral and bilateral proptosis in adults. Pseudo-ptosis and true ptosis may be seen in patients with TAO. The former results from contralateral lid retraction but true ptosis occurs when the levator muscle suffers dehiscence or when concurrent myasthenia gravis is manifested. Strabismus is common in TAO, resulting from restrictive extraocular muscle impairment. It can induce head tilt and diplopia. The inferior and medial rectus muscles are most commonly involved in TAO, resulting in horizontal and vertical deviations. The basis for this predilection has not been identified [23]. Diplopia develops from inflammation and swelling of the extraocular muscles and is generally restrictive. It is classified as intermittent (present upon awakening or during fatigue, present at extremes of gaze) or constant when present in primary gaze and/or reading position [4, 9]. One other important sign of EOM involvement is the elevation of IOP in upgaze, due to the restrictive action of the fibrotic inferior rectus muscles and blockade of the episcleral aqueous outflow. Orbital congestion also contributes to this elevation IOP [34, 35]. Attention should be paid to the position of the eye during applanation tonometry, which must be performed in the standard position and in down-gaze [36]. Upper-eyelid retraction (Dalrymple sign), often with temporal flare and scleral show, is one the most common ocular signs of TAO and should be differentiated from proptosis. Lid lag on down-gaze (von Graefe sign) is another important feature of the disease, manifesting as a downward saccadic movement with reduced amplitude [24]. Anterior segment signs in TAO include superficial punctate keratitis, superior limbic keratoconjunctivitis, conjunctival injection usually over the rectus muscle insertions and chemosis. Severe proptosis can cause corneal ulceration.

Vision disturbances in severe TAO may occur due to compressive optic neuropathy or dysthyroid optic neuropathy (DON). DON is defined as impairment of optic nerve function due to compression [37]. It presents as blurred vision, visual loss, dyschromatopsia, or field loss and can occur in up to 5% of patients with TAO [25, 38]. Visual impairment in TAO, resulting from dysfunction of the optic nerve, is caused by raised intraorbital pressure due to inflammation [39, 40]. Patients with optic nerve compression may not exhibit marked proptosis, but these individuals usually show substantially increased resistance to retropulsion. In addition, most cases of DON occur without visible optic nerve edema, making frequent documentation of visual acuity, color vision, and pupillary light reflex essential [4]. Due to orbital congestion, choroidal folds may also be seen in TAO, among other warning signs. These include corneal opacity, important lagophthalmos, and pale, swollen optic discs, which can signal impending DON [37]. Therefore, these are important to detect when evaluating patients with TAO. DON is the most serious quality of life-threatening condition associated with TAO [41], and requires immediate treatment [37].

## The ocular surface in TAO

A frequently underappreciated casualty of TAO is the ocular surface, a functional unit comprising the corneal and conjunctival epithelium, lid margins and tear film. Classically, increased palpebral fissure width and lid alterations caused by TAO have been implicated in the disruption of ocular surface homeostasis. This leads to corneal exposure, tear film instability, accelerated tear evaporation and high tear osmolarity [27, 28]. Eventually, ocular surface inflammation ensues, initiating a vicious cycle which eventually leads to dry eye syndrome (DES) [29].

Exophthalmos, with the resulting increased fissure width, lagophthalmos, and poor Bell’s phenomenon can contribute to DES. Inflammation of the OS and dry eye are frequently associated with TAO, sometimes preceding ophthalmic changes [42, 43].

In one report, patients with occult TAO consistently reported symptoms of ocular irritation, including foreign body sensation, redness, and excessive tearing [7]. These individuals were found to have OS inflammation in the absence of exophthalmos, lid retraction, dysmotility, and diplopia. Thus, the earliest forms of TAO may be confined to the OS, well in advance of lid retraction and lid lag.

A significant correlation was found between TAO activity, measured by CAS, and OS damage, detected by lissamine green staining [44]. In that study, the prevalence of dry eye was 65% in patients with TAO, and histopathologic changes in the conjunctiva were consistent with dry eye syndrome.

Gupta et al. [7] detected conjunctival and episcleral inflammation localized over the extraocular muscles in their entire series of patients and considered it to represent a presenting sign of TAO. Subtle widening of the inter-palpebral fissure was found in 48%, meibomian gland dysfunction in 48%, and a decreased tear break-up time (TBUT of less than 10 s) in 31% of these patients. Corneal and conjunctival vital staining, indicators of ocular surface damage, are a frequent sign in patients with TAO [7, 19, 28, 45].

Patients also present with reduced tear break-up time [19, 28], which indicates greater tear film instability. The Schirmer test, which assesses basal and reflex aqueous tear film production, may be normal [28] or reduced [19].

## Clinical diagnosis of DES in TAO

Diagnosis of DES can be made using simple, minimally invasive tests that are routinely conducted as part of the ophthalmological examination. These include administration of a questionnaire that assesses symptoms of ocular irritation and environmental triggers, such as the OS disease index (OSDI). TBUT, a procedure involving the instillation of fluorescein on the ocular surface, measures tear stability and exhibits the greatest correlation with other tests for DES diagnosis [44]. The Schirmer test, detects aqueous deficient dry eye with good sensitivity [46]. Fluorescein and lissamine green staining detect de-epithelized and devitalized ocular surface areas, respectively [45].

## TAO, lacrimal gland and the ocular surface

Lacrimal gland (LG) involvement in TAO may result from the direct effects of TSI, since acinar cells of the LG express TSHR [47]. Thyroid, salivary and lacrimal glands resemble one another histologically [48]. Further, all are particularly susceptible to immunological damage [49]. Sjögren’s syndrome (SS), an autoimmune disease characterized by chronic lymphocytic infiltration of LG and salivary glands [50], frequently affects patients with thyroiditis [49]. Histopathologic lesions in both diseases are infiltrated by T cells [51]. Patients with SS have a 74-fold greater chance of developing GD than the general population [52]. In TAO, TNF-α increases Fas expression on lacrimal cells, resulting in apoptosis and release of a fragment of α-fodrin [53].

Proteomic analysis of tear film can inform pathology occurring within lacrimal glands [54, 55]. Protective factors such as proline-rich proteins (PRPs) and cystatins were markedly down-regulated in patients with TAO compared to healthy individuals and those with DES [56]. Altered regulation of proinflammatory and protective proteins found in tears may reflect an inflammation-induced dysfunction of the LG in TAO [55]. Tear proteins were markedly different in those with TAO versus other forms of DES. These include proteins involved in inflammatory response, cell-to cell signaling and interaction, cellular motility and cell death. These findings suggest that different mechanisms induce LG and OS alterations in TAO [56].

Levels of IL-1β, IL-2, IL-6, IL-8, IL-10, IL-17, TNF-α and INF-γ were higher in tears from patients with active vs stable TAO. Further, cytokine levels generally correlated with CAS scores and fluorescein staining [19].

Direct autoimmune targeting in active TAO may contribute to the ocular surface disease, as is evidenced by detection of cytokines in tears [19] and active keratocytes, a putative biomarker for OS inflammation [57], respectively.

## Diagnostic considerations

The frequency and severity of TAO may be lessening in newly diagnosed GD hyperthyroidism. Further, TAO rarely progresses to more severe disease [58]. On the other hand, some patients, especially those exposed to tobacco, continue to present with severe disease and others manifest reactivation. Even in the absence of clinical ocular manifestations, imaging reveals subtle orbital changes in most patients with GD [4]. In nearly 70% of asymptomatic, hyperthyroid adults with GD, magnetic resonance imaging (MRI) and computed tomographic (CT) scanning reveal extra-ocular-muscle enlargement [9].

The most frequent clinical features of TAO are upper eyelid retraction, periorbital edema/erythema, and proptosis [9]. It is important to differentiate TAO from other common conditions that present similarly. These include orbital and pre-septal cellulitis, carotid cavernous fistula, orbital pseudotumor, and thickened muscles conditions such as sarcoidosis, neoplastic diseases and amyloid.

In most cases, the diagnosis of TAO can be established clinically. However, imaging studies may be required to evaluate orbit structures and aid in formulating an optimal treatment plan. It is possible to evaluate optic nerve compression on MRI and the orbital bony structure on CT. Neuroimaging usually reveals muscle enlargement with tendon sparing and fat expansion. Imaging may also reveal dilated superior ophthalmic veins and apical crowding of the optic nerve [59].

## Management of TAO

### General principles

Optimal care of patients with TAO requires a multidisciplinary approach. This usually includes both endocrinologists and ophthalmologists who typically provide primary care. Other specialists should participate as needed [60, 61]. Several academic centers have assembled multidisciplinary teams to facilitate treatment decisions and provide follow-up patient care, education, and family support [60, 61]. This is true here at the University of Michigan.

Restoration and maintenance of the euthyroid state is essential for all patients with TAO since wide swings in thyroid function can negatively impact its course [4, 60, 62]. Anti-thyroid drugs and surgical thyroidectomy are extremely effective for managing hyperthyroidism. Radioiodine treatment confers a small additional risk of exacerbating TAO or provoking its development de novo, particularly in those who smoke tobacco and in patients with severe hyperthyroidism. Immunosuppressive therapies such as B cell depletion with rituximab may prove effective and should be overseen by a collaborating rheumatologist/clinical immunologist [60, 62].

Patients with GD must be given all the necessary resources and guidance to achieve smoking cessation, irrespective of their ocular disease status. It should be considered a primary goal in the therapeutic plan. Exposure to smoke represents the single most modifiable environmental risk factor thus far identified for TAO [4, 60, 61]. Although the mechanisms responsible for its negative impact are not completely clarified, studies suggest that oxidative stress might represent the culprit, by inducing the expression of fibrosis-related genes and the increase of intracellular pro-inflammatory cytokines [63]. Smoking can lead to progression of TAO, smokers generally have more severe disease, and immunosuppressive treatment is typically less effective in smokers [60, 62, 64, 65]. Advanced age of onset, duration and severity of thyrotoxicosis, and smoking are risk factors [66]. Treatment with anti-thyroid medication was negatively correlated with developing TAO but smoking increased statistically the odds for the disease. Older patients with restricted ocular motility, strabismus, and active TAO are at higher risk of DON and may benefit from early medical intervention [66].

Selenium supplementation may provide benefit for mild cases of TAO; some patients experience improved quality of life and reduced eye symptoms [67, 68]. One study reported a positive effect in mild disease after a 6-month exposure to 100 mcg daily dosage [67]. Limitations of that study included a failure to analyze the background dietary intake of selenium and to determine whether the subjects in the study geographic regions were depleted of the element [60, 67, 68]. Another study failed to detect a correlation between decreased serum Selenium levels and increased TAO severity [69].

Treatments for DES and ocular surface disease in TAO should be personalized. Baseline treatment consists of artificial tears, moisturizing ointments, and supportive measures such as moisture chamber glasses, humidifiers, and protection from wind and smoke [60]. Topical anti-inflammatory therapy may prove beneficial in ocular surface disease.

## Therapy of active, moderate to severe TAO

Active TAO typically follows a 2 to 3-year course following Rundle’s curve that includes inflammatory signs, progression, and becomes “static at a level of incomplete recovery” [33]. Depending on its severity, active TAO can be followed with conservative measures [61]. Ocular surface lubrication must be preserved and artificial tears, gels and topical cyclosporine may be useful [70]. Topical treatment may prove insufficient to ensure corneal protection. In that case, lacrimal punctum occlusion or temporary tarsorrhaphies may become necessary [61, 71].

Glucocorticoid (GC) therapy is well established, although its benefits remain unproven in large prospective studies. The EUGOGO guidelines recommend prophylaxis of 0.3-0.5 ml prednisone/kg body weight in those undergoing radioiodine ablation of the thyroid who are at high risk of progression or de novo development of TAO. Lower risk patients may receive reduced GC doses [60]. GCs continue to be the first-line treatment of moderate-to-severe active TAO with unpredictable results. The recommended cumulative dose of intravenous GCs should not exceed 8.0 g (4.5 g as intermediate-dose and 7.5 g as a high-dose regimen for the worst cases) with carefully controlled diabetes and hypertension [60]. In special situations such as hepatic dysfunction, cardiovascular morbidity or psychiatric disorders, intravenous GCs should be avoided. Patients with severe reduction of visual acuity, visual field deficits, color desaturation or afferent pupillary defects are at risk for DON and must be treated promptly with high-dose systemic corticosteroids. In these cases, EUGOGO recommends intravenous methylprednisolone 500-1000 mg for 3 consecutive days or alternate days during the first week [60]. Should this prove ineffective, emergency orbital decompression surgery may become necessary [61]. GC can be administered orally, intravenously, or locally injected into the orbit [62].

Efficacy of intravenous and oral GCs were compared in moderately severe TAO patients; parenteral steroids were more effective in reducing CAS by at least 3 points, improvement in visual acuity, and decreasing disease activity at 3 months [62, 65]. In another study, GCs induced complete visual recovery in DON, improved visual acuity, color sensitivity, and normalized visual field defects after 2 weeks of treatment [72]. Combined parenteral and oral GCs were effective with a low rate of side-effects [73]. Intravenous administration appears more efficacious and is better tolerated than orally administered GCs [65, 74].

Radiation therapy (RT) has been reassessed recently in combination with GCs and was found to improve symptoms more than GCs alone [75]. While those results were promising, a controlled study will be necessary.

The effectiveness of alternative therapies for TAO is being studied, frequently in pilot, inadequately powered studies. B and T cell depletion, insulin-like growth factor-1 (IGF-I) receptor blockers, TSHR antagonists, and various cytokine antagonists are under scrutiny [76]. Rituximab (RTX), a monoclonal antibody recognizing targeting CD20^+^ B cells, has undergone pilot clinical trials with promising results in some studies [77, 78], while another suggests no benefit [79]. In an uncontrolled study of GC-resistant patients, RTX appeared to have benefit [77]. Larger multicenter trials will be necessary to establish the efficacy of RTX in TAO.

## Potential for IGF-I receptor inhibition as therapy for TAO

TSHR involvement in the pathogenesis of GD is well established, although clarifying its role in TAO remains to be accomplished. IGF-IR is over-expressed by orbital fibroblasts, T cell and B cells in GD, and thus may also participate in the disease [78, 80–83]. Both TSHR and IGF-IR appear to be activated by immunoglobulins that have been detected in GD (GD-IgGs). Tsui et al. [81] reported that crosstalk between TSHR and IGF-IR is critical to the downstream signaling initiated by TSHR activation. Fibrocytes express even higher levels of TSHR than do orbital fibroblasts [84, 85]. A very recent study confirmed the cross-talk occurring between IGF-IR and TSHR [82]. Activating anti-IGF-IR antibodies have been detected in some studies but not in others, leaving these concepts controversial [80, 81, 85]. Teprotumumab, an IGF-IR blocking monoclonal antibody, attenuates the induction by TSH and TSIs of cytokines in fibrocytes [84]. The antibody has been examined for its potential therapeutic benefits in a multicenter, placebo-controlled clinical trial of active, moderate to severe TAO [http://clinicaltrials.gov/show/NCT01868997]. Results from this study should be available in the next few months.

Cytokines represent potentially important therapeutic targets in active TAO [86]. Tocilizumab, a recombinant, humanized monoclonal antibody that antagonizes the IL-6 receptor, was administered intravenously to eighteen patients with TAO who had proven refractory to intravenous GC in an uncontrolled trial [87]. Improvement of CAS was observed in all subjects, proptosis decreased in 72%, and ocular motility improved in 83.3%. No severe side effects or relapses of active TAO were observed at the end of a follow-up period of at least 9 months. One patient with compressive optic neuropathy improved, avoiding orbital decompression. Further studies involving well-controlled, randomized and masked trials of this and other anti-cytokine candidates will be necessary in determining whether these approaches might be effective.

## Remediation in stable TAO

Most surgical treatments for TAO are reserved for inactive disease. The notable exceptions are active cases which require urgent orbital decompression surgery for DON or sight-threatening optical surface damage. Once the stable phase has been reached, treatments are largely surgical, aiming at anatomic, functional, and cosmetic rehabilitation. Surgeries are typically staged and planned individually, depending on dysfunction and disfigurement [61]. Decompression surgery, strabismus surgery, lid lengthening and cosmetic periorbital surgeries, may be required. These should follow this particular sequential order since the outcome of each procedure may determine the necessary goals of the next [60].

**Fig. 1:**
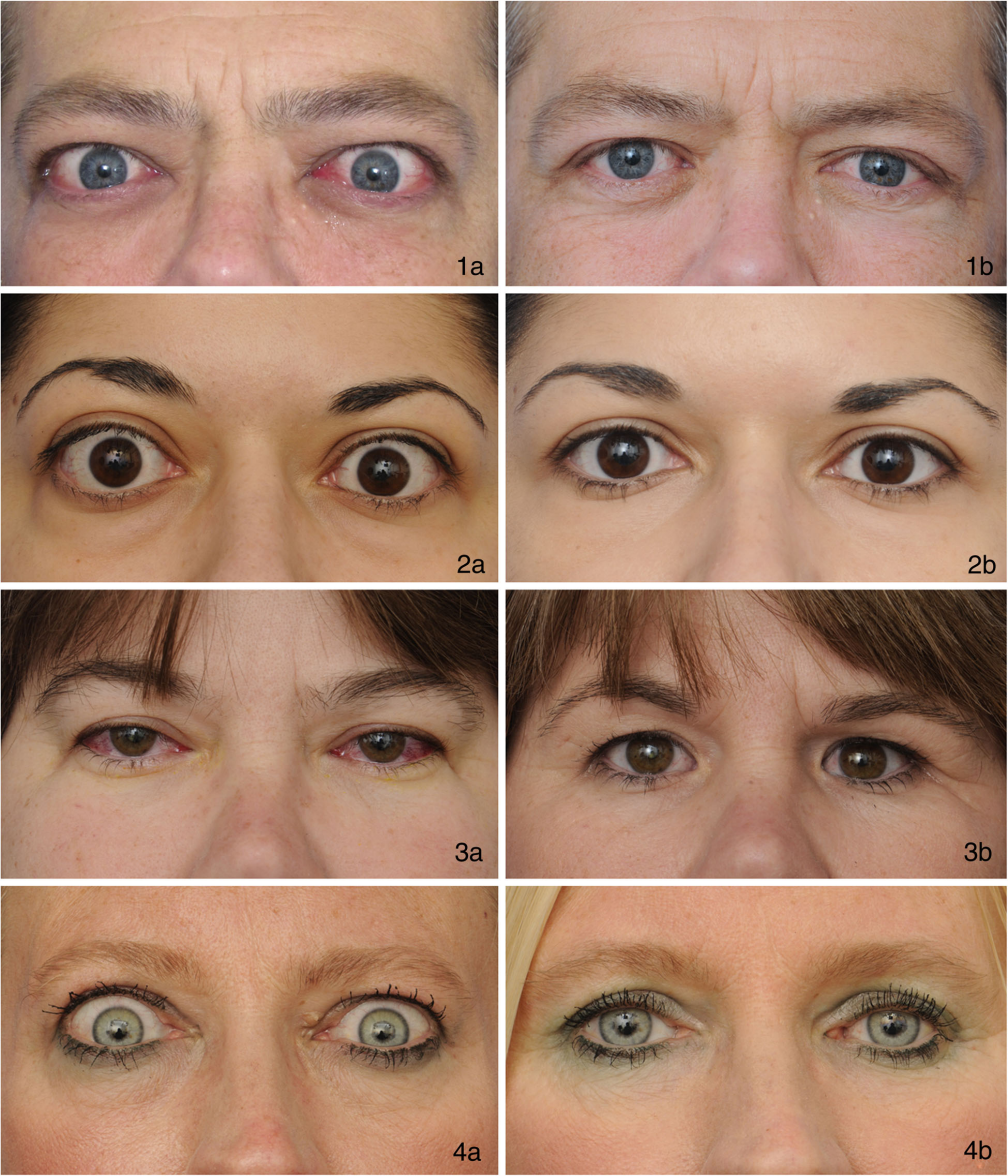
Patients with thyroid-associated ophthalmopathy before (1**a**, 2**a**, 3**a**, 4**a**) and after (1**b**, 2**b**, 3**b**, 4**b**) surgical treatment. These images exemplify the most common signs of ophthalmopathy, including proptosis, conjunctival hyperemia, periocular edema and upper and lower eyelid retraction. These may improve with treatment. These images were generously provided by Dr. Raymond Douglas, Kellogg Eye Center, University of Michigan, Ann Arbor, USA﻿

Different decompression techniques have been developed. Their use should be tailored to the specific therapeutic goals of each case. Bone and fat removal may be performed separately or combined to maximize decompression. Modern approaches include infero-medial wall, lateral wall, and combined (balanced) decompressions. In general, the appropriate decompression procedure is one that will result in the degree of proptosis reduction that is sought [4] (Fig. 1). Lateral and medial wall approaches offer both advantages and drawbacks. For instance, lateral wall decompression is accompanied by less post-operative strabismus but a longer convalescence period. Further, medial wall procedures can frequently accomplish greater proptosis reduction [88]. Strabismus/diplopia may be worsened by decompression and thus may require additional intervention. Minimally invasive approaches have been advocated by some [60, 89]. Endoscopic techniques may allow decompression with less morbidity, accessing areas with good visibility and less exposure. Purely endoscopic procedures and intraoperative surgical tailoring with personalized boney decompression have resulted in good outcomes [90]. These procedures can reduce intraocular tension and provide pain relief, improve strabismus and correct postural visual obscuration in patients with orbital and optic nerve microvasculopathy [90]. The most common surgical complications include de novo onset or worsening of preexisting strabismus and globe dystopia [89, 91]. Despite normalization of visual acuity and resolution of optic nerve head edema, almost half of patients with substantial nerve damage will manifest persistent visual field defects following adequate decompression [90]. On the other hand, improvement in severe vision loss as late as 3-month after onset has been reported following decompression, suggesting that the procedure may be effective in reversing DON in patients with NLP vision [92].

Criteria with which to judge success of strabismus surgery in TAO are poorly defined. These procedures lack standardization, making surgical outcomes difficult to compare. GO-QoL may be useful in assessing surgical outcomes [93]. At least one study demonstrated improved GO-QoL score following strabismus surgery in TAO [94].

Upper eyelid retraction is the most common clinical sign in TAO. It is frequently improved but rarely completely corrected following orbital decompression [62, 71]. Several techniques have been developed for correcting upper eyelid retraction. These aim at weakening retractor muscles, by recession, partial resection, or lengthening [24]. Surgical outcomes in upper eyelid repair are difficult to predict and many different techniques have emerged yielding variable results [24, 62]. Lower eyelid retraction may also be surgically corrected; however, no consensus as to the best approach has been reached. The surgeon’s preferences and expertise, anatomical variations, outcome expectations, attitude towards intervention, and disease severity should guide the choice of surgical method [91].

## Conclusions

Nearly two hundred years after the first descriptions of GD, we continue to discover more about TAO, its molecular underpinnings, clinical behavior, and attempt to identify improved therapies. Advancing research techniques have led us to clearer insights into this vexing disorder. But substantial barriers remain, including the absence of proven animal models possessing the necessary fidelity to human disease, better access to affected tissue, and more aggressive organization of large, multicenter clinical trials. Ultimately, our goal must focus on restoring immune tolerance to the autoantigens that underlie the disease. That approach will spare many patients the adverse effects of chronic immune suppression and the invasive surgical approaches currently employed.

## Abbreviations

CAS: Clinical activity score
CD34: Hematopoietic cell antigen CD34
CPF: Capsulopalpebral fascia
CT: Computed tomography
DES: Dry eye syndrome
DON: Dysthyroid optic neuropathy
EOM: Extraocular muscle
Fas: Apoptosis antigen 1 (CD95)
GC: Glucocorticoid
GD: Graves’ disease
GO-QoL: Graves’ ophthalmopathy quality of life questionnaire
IGF-1R: Insulin-like growth factor-1 receptor
IL-1β: Interleukin 1 Beta
IL-2: Interleukin 2
IL-6: Interleukin 6
IL-8: Interleukin 8
IL-10: Interleukin 10
INF-γ: Interferon gamma
IOP: Intraocular pressure
LG: Lacrimal gland
LPS: L*evator palpebrae superioris* muscle
MRI: Magnetic resonance imaging
NLP: No light perception
OCT: Optical coherence tomography
OS: Ocular surface
OSDI: Ocular surface disease index
POTEI: POTE Ankyrin domain family member I
PRPs: Proline rich proteins
RNFL: Retinal nerve fiber layer
RT: Radiation therapy
RTX: Rituximab
SS: Sjögren’s syndrome
TAO: Thyroid-associated ophthalmopathy
TAO-Igs: Thyroid-associated ophthalmopathy immunoglobulins
TBUT: Tear break-up time
Thy-1: Thymocyte antigen 1
TNF-α: Tumor necrosis factor alpha
TSHR: Thyrotropin Receptor
TSI: Thyroid stimulating immunoglobulins
